# Understanding participation and quality of life of university students with probable DCD using structural equation modeling

**DOI:** 10.1016/j.bjpt.2026.101658

**Published:** 2026-10-05

**Authors:** Miri Tal-Saban, Naomi Weintraub, Hagit Magen

**Affiliations:** School of Occupational Therapy, The Hebrew University of Jerusalem, Israel

**Keywords:** Developmental coordination disorder (DCD), Engagement, Higher education, Motor difficulties, Well-being, Young adults

## Abstract

**Backgrounds:**

Individuals with developmental coordination disorder (DCD) attending higher-education institutions face challenges in their everyday life. Nevertheless, research regarding participation and quality of life (QoL) in this population is limited.

**Aim:**

To explore determinants of participation and QoL in university students with probable DCD (pDCD) through structural equation modelling (SEM).

**Methods:**

A cross-sectional comparative and correlational design. A random sample of 297 undergraduate university students was divided into three groups based on cutoff scores of the Adolescents & Adults Coordination Questionnaire: pDCD; *n* = 40, borderline pDCD (BpDCD) *n* = 45 and typically developing (TD) *n* = 212. Measures included self-report questionnaires relating to pDCD, attention deficit and hyperactivity disorder (ADHD) symptoms, executive functions (EF), including metacognition and behavioral regulation, as well as participation and QoL.

**Results:**

Students with pDCD reported reduced participation and QoL and impairment in EF and ADHD compared to TD students. SEM results showed that pDCD had a direct significant effect on EF, ADHD symptoms and participation and an indirect effect on QoL, mediated most strongly by participation. ADHD and behavioral regulation had a direct effect on QoL, whereas the relationship between metacognition and QoL was mediated by participation.

**Conclusions and relevance:**

Through a SEM model, this study demonstrated that pDCD in university students is associated with reduced participation and QoL, and emphasized the role of EF, ADHD and participation in explaining QoL. These findings offer important insights for the assessment and intervention of higher-education students with pDCD.

## Introduction

The number of individuals with neurodevelopmental disorders, including developmental coordination disorder (DCD), enrolling in higher education has gradually increased.^1^^,^^2^ Occupational therapists play a key role in assessing these students’ unique needs, and providing specialized interventions that maximize their potential for full participation in diverse activities, thereby enhancing their quality of life (QoL).^3^^,^^4^ DCD is a common chronic neurodevelopmental disorder defined by four criteria^5^: (A) motor coordination difficulties while executing motor tasks; (B) significant interference with activities of daily living, affecting academic/school achievement, leisure, and play; (C) onset during early development; and (D) symptoms are not due to intellectual disability or neurological impairments. When one or more of these criteria have not been formally evaluated, individuals may be considered as having probable DCD (pDCD).^6^

Although the prevalence of DCD in adults is not known, a study by Gabbard et al.^2^ reported that 7% of higher education students were found to have pDCD. However, various studies reported gender differences in the exhibition of DCD symptoms. For example, females reported more difficulties in gross motor and non-motor related daily activities, whereas males reported more difficulties in daily activities related to fine motor.^2^ Furthermore, DCD has been linked to various co-occurring neurodevelopmental disorders,^5^^,^^7^ with the highest co-occurrence rate of over 50% with attention deficit and hyperactivity disorder (ADHD).^8^^,^^9^ DCD is increasingly recognized as involving more than motor impairments, with evidence of accompanying executive functions (EF) deficits.^10^^,^^11^ EF refers to high-level cognitive processes essential for goal-directed behavior, such as inhibition, planning, and working memory.^12^ EF deficits may hinder motor adjustments, and consequently overall task performance.^11^^,^^13^ In adults with DCD, EF and motor difficulties are associated with participation restrictions and reduced QoL.^10^

Participation, defined as "involvement in activities in all life domains", is often expressed in frequency and quality of activity performance.^14^ Participation has been emphasized as a key component of achieving high QoL.^14^ The WHO defines QoL as "individuals' perception of their position in life, in the context of the culture and value systems in which they live, and in relation to their goals, expectations, standards and concerns".^15^ Both participation and QoL are regarded as main health outcomes.^14^^,^^16^

One area in which young adults with DCD may be particularly affected is higher education.^17^ In addition to the new responsibilities of young adulthood, such as independent living and financial management,^18^^,^^19^ students with DCD often encounter difficulties in higher education due to motor and EF deficits. These impairments can impede their ability to meet both academic and daily life demands,^17^^,^^20^ limiting their participation in educational settings. In fact, many students with DCD continue to live with their parents, suggesting broader challenges in achieving independence and full participation.^21^ Consequently, the demands of university life place students with DCD at increased risk of participation restrictions and reduced QoL.

Despite the potential severity of these challenges, research on participation and QoL of higher-education students with DCD or pDCD remains limited.^21^ To our knowledge, no previous studies have examined the interrelationships between pDCD, EF, ADHD, participation and QoL. This study had several objectives: (a) to describe the participation and QoL of university students with pDCD compared to their typically developing (TD) peers; (b) to describe a statistical model (using structural equation modeling; SEM), to understand the challenges faced by university students with pDCD in their daily participation and QoL, and the contribution of deficits in EF and ADHD to the relationship between pDCD and participation and QOL (see Figure A.1 in Supplementary material).

## Methods

### Participants

A total of 297 undergraduate university students participated in this study. Students were invited to participate in the study, and were excluded from the study only if they reported having mental health problems (e.g., PTSD, Major Depressive Disorder) or a physical disorder that may affect their daily functioning. Twenty-one students were excluded from the study due to these health problems. However, students who reported having ADHD were not excluded. Three groups were identified based on the Adolescents & Adults Coordination Questionnaire (AAC-Q)^22^ that provides cutoff scores for pDCD and borderline pDCD (BpDCD). Forty students were identified with pDCD (32 female; *M*_age_ = 23.85, *SD* = 2.75), 45 students were identified as BpDCD (33 female; *M*_age_ = 22.86, *SD* = 2.21), and 212 students were assigned to the TD control group (154 female, *M*_age_ = 23.01, *SD* = 2.93). The three groups did not differ in age *F*(2, 283) = 1.62, *p* = 0.20, *η_p_*^2^ = 0.011 or in gender distribution *χ*^2^(2) = 0.95, *p* = 0.62.

### Measures

#### *The Adolescents & Adults Coordination Questionnaire (AAC-Q)*^22^

The AAC-Q is a 12-item self-report questionnaire that addresses motor-related everyday activities, screening for motor coordination deficits among adolescents and young adults. Items are rated on a Likert scale from 1 (Never experience difficulty) to 5 (Always). The total sum score ranges from 12 to 60. The AAC-Q demonstrated good reliability and validity, employing cutoff scores to identify pDCD (score>32, above 1.65 SD) and BpDCD (score=27–32, 1–1.65 SD, corresponding to the 5th–15th percentiles). The AAC-Q was used to assign participants to one of three groups: pDCD, BpDCD, or control. In this study Cronbach's alpha for the AACQ was 0.72.

#### *Wender- Utah Rating Scale (WURS)*^23^

The WURS is a 61-item self-report questionnaire designed to assess the presence and severity of childhood ADHD symptoms in adults. This study used the 25 items shown to effectively differentiate adults with and without ADHD.^24^ Items are rated on a 5-point Likert scale (0 = not at all or very slightly to 4 = very much), and the total score is the sum of all items. A score above 46 suggests possible ADHD.^23^ The WURS has acceptable test-retest reliability, internal consistency and concurrent validity, and has shown to distinguish between adults with and without ADHD.^25^ In this study Cronbach's alpha for the WURS was 0.92.

#### *Participation in Everyday Activities of Life (PEDAL)*^26^

The PEDAL is a 14-item self-report ecologically valid questionnaire that assesses participation of adolescents and young adults in a range of daily activities requiring coordination, organization and planning. Items are scored on a 5-point Likert scale, from 1 (always) to 5 (never). The total score is calculated by summing up all items. A higher score indicates lower participation. The PEDAL has demonstrated good internal consistency and validity. In this study Cronbach's alpha for the PEDAL was 0.58.

#### *The World Health Organization Quality of Life instrument (WHOQOL-BREF)*^15^

The WHOQOL-BREF is a valid self-report 26-item shortened version of the original WHOQOL-100. It measures four domains: Physical health, Psychological health, Social relationships, and Environment. Two additional questions evaluate general QoL and health. Items are scored on a 5-point Likert scale, from 1 (not satisfied) to 5 (very satisfied). The four domain scores are calculated by summing up items related to each domain. Higher scores indicate greater QoL. In this study Cronbach's alpha for the WHOQOL-BREF was 0.83.

#### *Behavior Rating Inventory of Executive Function- Adult version (BRIEF-A)*^27^

The BRIEF-A is a 75-item, reliable and valid self-report questionnaire that assesses everyday behavioral manifestations of EF in adults. It includes nine scales: Inhibit, Shift, Emotional Control, Self-Monitor, Initiate, Working Memory, Plan/Organize, Task Monitor and Organization of Materials. The first four scales form the Behavioral Regulation Index (BRI), and the remaining scales form the Metacognition Index (MI). The Global Executive Composite (GEC) is the overall score of both indices. Items are scored on a 3-point Likert scale from 1(never) to 3 (often), and are converted to standard T scores normed by age and gender. Lower scores indicate better EF. In this study Cronbach's alpha for the BRI was 0.88 and 0.93 for the MI.

Because all the instruments were self-report, we conducted the Harman's single-factor test to assess common method bias. We found that the total variance explained when using a single factor was 18.25%, which is lower than the acceptable 50% threshold. This analysis indicates that a common method bias is unlikely to be a significant issue in these data**.**

### Procedure

Approval to perform this study was granted by the Hebrew University ethics committee (#12,032,018). Participants were recruited through advertisements posted around campus. Participants signed an informed consent and completed the questionnaires in the presence of the researcher who was available to answer questions. Participants received course credit or payment for their participation.

### Data analysis

Statistical analyses were performed using the Statistical Package for Social Sciences for windows (SPSS version 29, IBM Corp., New York). Group differences were analyzed using multivariate and univariate analyses of variance (MANOVA\ANOVA), with planned contrasts comparing the scores between the TD and the BpDCD groups, and between the BpDCD and the pDCD groups. Post-hoc contrasts, using the Bonferroni correction for multiple comparisons compared the TD and the pDCD groups.

Structural Equation Modeling was performed using IBM AMOS Graphics software (Version 29, IBM Corp., New York) to test the direct effects of pDCD on the study variables of EF (separately for the Behavioral regulation and Metacognition indices), ADHD and participation, and on the dependent variable QoL. The hypothesized model was tested using maximum likelihood estimation, and bootstrapped 95% confidence intervals were calculated based on 5000 re-samples. Goodness-of-fit was assessed through Chi-square, Comparative Fit Index (CFI), Tucker-Lewis Index (TLI) and Root-Mean Square Error of Approximation (RMSEA). A non-significant chi-square, and CFI ≥ 0.95, TLI ≥ 0.95, RMSE ≤ 0.08 were taken as indicators of an acceptable fit.^28^ The data of the pDCD and BpDCD groups were combined to yield a single pDCD group, after verifying that the model for each group yielded similar results. Additionally, given potential gender-related effects and the higher number of female participants, gender was entered into the SEM as a covariate. Pearson correlations were used to further examine the associations between study variables and the four domains of QOL. Type I error of 0.05 was used for all statistical analyses.

## Results

### Group differences in study measures

In the first analysis, the three groups of participants were compared on the nine scales of the BRIEF-A (see Table 1). The MANOVA revealed a significant overall Group effect, *F*(18,570) = 8.27, *p* < 0.001, Wilk’s Λ = 0.629, *η_p_*^2^ = 0.207, and significant Group effects in all nine scales of the BRIEF-A. Planned contrasts showed significantly higher scores on all nine scales in the BpDCD and the pDCD groups compared to the TD group (Table 2), suggesting lower EF. The pDCD group scored significantly higher than the BpDCD groups in six of the nine scales, mainly those falling under the Metacognition domain (Table 2).

**LTable 1:** Means, Standard Deviations and MANOVA Results of Group Differences in the BRIEF-A T Scores, and WHOQOL Scores.

	Control^a^		BpDCD		pDCD		MANOVA results	
BRIEF-A (T score)	Mean	SD	Mean	SD	Mean	SD	*F*(2293)	*η_p_*^2^
Inhibit	47.84	7.47	53.00	8.32	55.63	9.78	20.76	0.124⁎⁎⁎
Shift	53.91	9.71	58.16	8.24	62.45	9.14	15.55	0.096⁎⁎⁎
Emotional control	49.41	8.79	53.60	8.36	54.80	9.58	8.98	0.058⁎⁎⁎
Self-monitor	45.63	7.48	49.78	7.01	50.92	9.70	11.34	0.072⁎⁎⁎
Initiate	49.51	9.45	54.36	9.78	59.27	9.73	19.89	0.120⁎⁎⁎
Working memory	51.90	9.24	59.98	9.57	68.72	12.67	55.16	0.274⁎⁎⁎
Plan/Organize	49.28	7.50	55.87	9.08	63.57	10.33	56.63	0.279⁎⁎⁎
Task monitor	50.47	9.58	58.58	8.79	64.48	12.03	40.89	0.218⁎⁎⁎
Organization of materials	45.71	8.43	52.11	8.77	57.22	9.40	35.35	0.194⁎⁎⁎
BRI	49.03	7.83	54.40	6.34	56.98	8.61		
MI	49.10	7.80	56.96	8.32	64.33	10.23		
GEC	49.04	7.63	56.27	7.01	62.18	8.57		
WHOQOL-BREF								
Physical Health	56.31	10.00	53.13	11.58	48.55	12.05	9.75	0.062⁎⁎⁎
Psychological Health	68.65	10.45	65.18	8.23	60.85	10.32	10.89	0.069⁎⁎⁎
Social Relations	56.30	13.09	55.49	14.37	55.15	15.00	< 1	0.001
Environment	73.00	11.64	69.00	11.87	63.55	12.33	11.71	0.074⁎⁎⁎

⁎⁎⁎ *p* < 0.001.

a Data of 1 participant from the control group was missing in the two analyses. BRI=Behavioral regulation index; MI=Metacognitive index; GEC=Global executive; WHOQOL=World Health Organization Quality of Life instrument.

**Table 2:** Planned Contrasts and Multiple Comparison (Bonferroni Correction) Tests on Group Differences in the BRIEF-A and WURS Questionnaires.

	Planned contrasts				Post-hoc	
	TD-BpDCD		BpDCD-pDCD		TD-pDCD	
BRIEF-A (T score)	Mean difference	*p*	Mean difference	*p*	Mean difference	*p*
Inhibit	−5.16	<0.001	−2.63	0.13	−7.78	<0.001
Shift	−4.24	<0.01	−4.29	<0.05	−8.54	<0.001
Emotional control	−4.19	<0.01	−1.20	0.53	−5.39	0.001
Self-monitor	−4.15	0.001	−1.15	0.50	−5.30	<0.001
Initiate	−4.84	<0.01	−4.92	<0.05	−9.76	<0.001
Working memory	−8.08	<0.001	−8.75	<0.001	−16.82	<0.001
Plan/Organize	−6.58	<0.001	−7.71	<0.001	−14.29	<0.001
Task monitor	−8.10	<0.001	−5.90	<0.01	−14.00	<0.001
Organization of materials	−6.40	<0.001	−5.11	<0.01	−11.51	<0.001
WHOQOL-BREF						
Physical Health	3.18	0.07	4.58	<0.05	7.76	<0.001
Psychological Health	3.47	<0.05	4.33	0.05	8.00	<0.001
Social Relations	0.82	0.71	0.34	0.91	1.16	1
Environment	4.01	<0.05	5.45	<0.05	9.45	< 0.001

WHOQOL=World Health Organization Quality of Life instrument;.

A one-way ANOVA on the WURS total score revealed a significant main effect of Group *F* (2290) = 18.72, *p* < 0.001, *η_p_*^2^ = 0.114. The WURS was significantly lower in the TD group (*M* = 18.15, *SD* = 12.18) compared to the BpDCD group (*M* = 25.93, *SD* = 14.52) *p* < 0.001, CI 95% [−12.09: −3.48], and the pDCD group (*M* = 30.92, *SD* = 14.10) *p* < 0.001, demonstrating that participants with pDCD and BpDCD reported more severe symptoms of childhood ADHD compared to TD participants. The difference between the pDCD and the BpDCD groups was not significant, *p* = 0.09, CI 95% [−10.72: 0.74].

Further analyses compared the scores of the three groups on the PEDAL and the WHOQOL-BREF questionnaires. A one-way ANOVA on the PEDAL’s total score showed a significant main effect of Group *F*(2294) = 36.46, *p* < 0.001, *η_p_*^2^ = 0.199, with significantly lower scores in the TD group (*M* = 2.20, *SD* = 0.51) relative to the BpDCD group (*M* = 2.52, *SD* = 0.44) *p* < 0.001, CI 95% [−0.49: −0.16], and the pDCD group (*M* = 2.91, *SD* = 0.57) *p* < 0.001, suggesting reduced participation in both pDCD groups. The difference between the pDCD group and the BpDCD group was also significant *p* = 0.001, CI 95% [−0.60: −0.17], as the PEDAL was higher in the pDCD group.

A MANOVA based on the WHOQOL-BREF scores (see Table 1) revealed a significant overall Group effect, *F*(8580) = 4.43, *p* < 0.001, Wilk's Λ = 0.888, *η_p_*^2^ = 0.058, and significant Group effects in the Physical Health, Psychological Health and Environment domains, but not in the Social Relations domain. The TD group showed significantly higher scores compared to the BpDCD group in the Psychological Health and Environment scales, and significantly higher scores compared to the pDCD group in the Physical Health, Psychological Health and Environment domains (Table 2). The differences in these domains were also significant between the pDCD and the BpDCD groups, with higher scores in the latter.

### Factors contributing to students’ participation and QoL; SEM analysis

A path analysis was performed to examine the potential influence of pDCD, ADHD, EF and participation on QoL while controlling for gender (see Fig. 1). The model had a good fit to the data χ²_(27)_ = 34.07, *p* = 0.199, CFI = 0.993, NFI = 0.961, RMSEA = 0.027, and TLI = 0.985 (see Fig. 1).

**Fig. 1:**
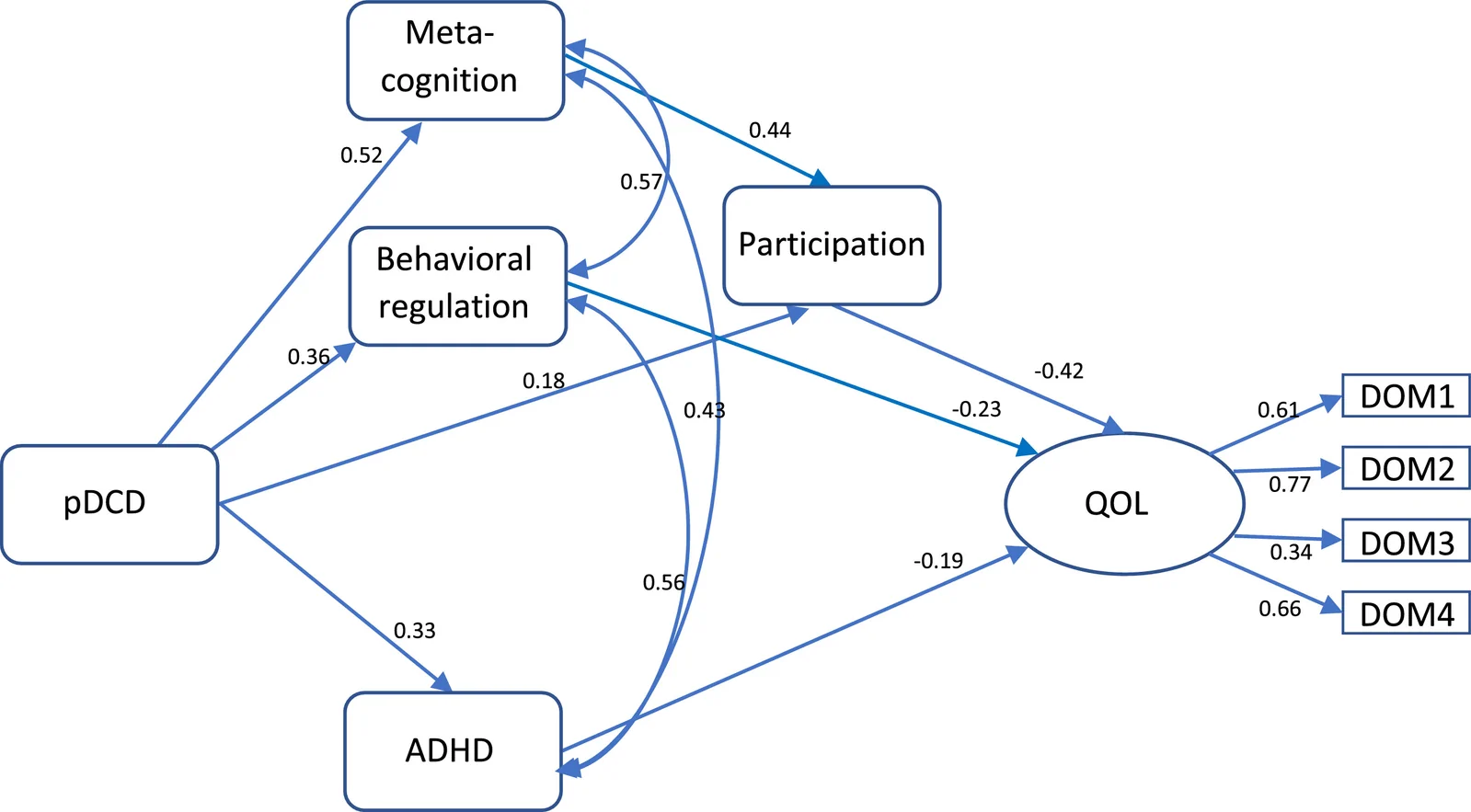
A Structural Equation Model (SEM) Analysis of the Effect of Probable DCD (pDCD and BpDCD), Executive Functions (Metacognition and Behavioral Regulation), Attention and Participation on Quality of Life^a^. All the Paths in the Model are Statistically Significant at p < 0.05. ^a^Gender was added to the model as a covariate. DOM1=Physical Health; DOM2=Psychological Health; DOM3=Social Relations; DOM4=Environment.

The model revealed that pDCD had a direct significant effect on all the variables in the model except for QoL, such that pDCD was associated with deficits in ADHD and EF, and with reduced participation. The model revealed additional indirect effects that were all significant (see Table 3). While pDCD did not directly impact QoL, it had an indirect effect on QoL via ADHD, the behavioral regulation index and participation, with participation showing the highest effect on QoL. Additionally, pDCD showed an indirect effect on participation via the metacognition index, suggesting that pDCD with more deficits in metacognition further contributed to reduced participation and subsequently to reduced QoL. Additional indirect effects of pDCD on QoL indicated that the association of pDCD with deficits in ADHD and behavioral regulation contributed further to decreased QoL, irrespective of the indirect effect of pDCD on participation.

**Table 3:** Indirect Effects on Quality of Life With 95% Bootstrapped Confidence Intervals.

Path	Indirect effect coefficient	p	95%CI
pDCD-> MC-> Participation -> QOL	−1.62	0.000	[−2.69, −0.87]
pDCD ->BR -> QOL	−1.48	0.001	[−2.75, −0.60]
pDCD -> Participation -> QOL	−1.21	0.001	[−2.29, −0.48]
pDCD -> ADHD -> QOL	−1.04	0.010	[−2.27, −0.27]
MC -> Participation-> QOL	−0.14	0.001	[−0.23, −0.08]

pDCD= probable DCD, MC = Metacognition, BR = Behavioral Regulation, QOL= Quality of Life, ADHD = attention deficit hyperactivity disorder.

Finally, an additional correlation analysis was conducted between the study variables and the four domains of QoL (see Table 4). The Bonferroni correction for multiple comparisons was applied with a threshold of 0.0025 (= 0.05/20 (correlations). After the application of the correction, the analysis revealed significant moderate correlations between the study variables and the QoL Physical health, Psychological health and Environment domains but not with social relations.

**Table 4:** Pearson Correlation Coefficients Between the Study Variables and the Four Domains of the QOL Measure.

Variables	AAC-Q	BRI	MI	WURS	PEDAL
WHOQOL–BREF					
Physical Health	−0.32⁎⁎⁎	−0.27⁎⁎⁎	−0.32⁎⁎⁎	−0.25⁎⁎⁎	−0.37⁎⁎⁎
Psychological Health	−0.32⁎⁎⁎	−0.38⁎⁎⁎	−0.38⁎⁎⁎	−0.40⁎⁎⁎	−0.42⁎⁎⁎
Social Relations	−0.08	−0.10	−0.17⁎⁎	−0.13*	−0.11
Environment	−0.35⁎⁎⁎	−0.35⁎⁎⁎	−0.30⁎⁎⁎	−0.27⁎⁎⁎	−0.39⁎⁎⁎

⁎ *p* < 0.05.

⁎⁎ *p* < 0.01.

⁎⁎⁎ *p* < 0.001 (two tailed *p* values, before applying the Bonferroni correction for multiple comparisons, see text for further details)*.* AAC-Q=Adolescents and Adults Coordination Questionnaire; BRI=Behavioral Regulation Index; MI=Metacognitive Index; PEDAL=Participation in Every Day Activities of Life; WHOQOL–BREF=World Health Organization Quality of Life instrument; WURS=Wender-Utah Rating Scale.

## Discussion

Higher education students with DCD encounter numerous challenges in participation in daily life, beyond those typically experienced by the general student population,^18^ and which is often associated with reduced QoL. However, the data relating to the factors contributing to reduced participation and QoL are limited. This study explored the contribution of deficits in EF and ADHD to the relationship between pDCD and participation and QoL in university students with pDCD through SEM.

First, the findings established that students with pDCD and BpDCD participated less in daily activities than their TD peers, consistent with previous studies among young adults with pDCD, which reported participation restrictions in academic, leisure and daily living activities (e.g., driving, organizing equipment^20^^,^^29^). Additionally, both pDCD groups reported lower physical and psychological QoL, but not social QoL. This finding contrasts the result of Engel-Yeger,^30^ but aligns with Tal-Saban and Kirby,^7^ who found no differences in social participation and relationships between young adults with pDCD or BpDCD and their TD peers. Moreover, Walker^31^ reported that young adults with pDCD identified social skills as a relative strength. Additionally, consistent with previous research, both pDCD groups reported significantly greater deficits in EF (BRI and MI) and ADHD than TD students,^26^^,^^32^ in line with the well-documented co-occurrence of DCD and ADHD.^5^

After determining group differences, we used SEM to examine how pDCD (including BpDCD), EF, ADHD and participation are associated with QoL in university students. Most notably, the analysis revealed that pDCD had a direct effect on all variables except QoL. The analysis showed that the relationship between pDCD and QoL was mediated by BRI and ADHD symptoms and by participation. These findings support prior research linking pDCD and EF and ADHD^9^^,^^17^ suggesting that DCD extends to cognitive deficits, beyond motor coordination deficits. Accordingly, Purcell et al.^33^ found that 30% of young adults with DCD primarily complained of EF difficulties and sought diagnosis and intervention due to these difficulties, rather than their motor problems.

Interestingly, the two EF components examined showed distinct effects. BRI, related to reward management, emotion regulation, and impulse inhibition, had a direct effect on QoL but not on participation. In contrast, MI, which involves planning, organization and working memory^34^ was indirectly related to QoL via the mediation of participation. Participation, measured by the PEDAL^26^ emphasizes organizational skills. Thus, these findings suggest that the reduced QoL in university students with pDCD may be partly explained by difficulties in organization-related skills such as time management and strategy use.

While the SEM indicated that EF (BRI) and ADHD mediated the relation between pDCD and QoL, the strongest association of pDCD and QoL was mediated through participation in daily activities. This suggests that motor coordination difficulties alone did not directly reduce QoL, but rather, it appears that participation restrictions might be the key mechanism. This finding aligns with Zaguri-Vittenberg et al.,^20^ who found that adults with DCD reported daily participation as effortful, frustrating, and stressful. This also supports the findings that reduced participation is negatively associated with QoL and well-being.^26^ Furthermore, Forde & Smyth,^35^ found that adults with DCD show elevated avoidance behaviors, particularly in social and occupational contexts. Importantly, these behaviors, not the severity of motor impairment, predict lower QOL. This indicates that reduced participation, driven by avoidance, may be the primary pathway through which DCD affects well-being. Engel-Yeger^36^ similarly demonstrated that motor difficulties affect QoL mainly through their emotional and cognitive consequences, as greater motor deficits were associated with higher emotional distress, lower self-efficacy, and poorer functional cognition, factors that, rather than motor severity itself, accounted for reduced QoL.

We believe that this is one of the first studies providing comprehensive data concerning the mediation effect of cognitive dysfunctions and participation restrictions on QoL of higher education students with pDCD. However, it is important to highlight several limitations. The pDCD students were identified solely based on self-reports rather than through motor assessments. This has been a common practice in studies on adults^2^^,^^37^ since there were no valid motor coordination assessment tools for adults. While the AACQ has been validated an assessment tool for pDCD, future studies should generalize the current results to a population of students identified by objective motor coordination assessments. Furthermore, the internal consistency of the PEDAL was lower than expected.^26^ However, given the low reliability, the significant associations found here may represent underestimates of the true effects. Replication with more reliable measurement would clarify the magnitude of these relationships. Additionally, the majority of the sample consisted of female students. Although this represents a noted gender bias in response to surveys among higher education students,^38^ it does not reflect the higher prevalence of males diagnosed with DCD,^5^ which may limit the external validity of this study. Nevertheless, to address this limitation, gender was introduced as a covariate in the SEM model. Future research should involve a more representative sample and include direct evaluations of DCD for more accurate diagnosis**.**

## Conclusion

This study contributes to the understanding of the associations of pDCD with participation and QoL of university students. Consistent with previous research, students with pDCD reported greater EF deficits (both BRI and MI), reduced participation, and lower QoL than TD peers. Participation, as well as EF and ADHD emerged as key mediators between pDCD and QoL, emphasizing the important role of occupational therapists in supporting higher-education students with pDCD, and enhancing their participation in daily life activities and QoL. Future research should further explore these relationships in larger, more diverse samples, and with direct assessments of motor coordination.

## Submission declaration

We declare that this manuscript is original, has not been published or submitted elsewhere, has been approved by all authors and relevant authorities, and will not be published in the same form without the copyright holder’s consent.

## Declaration of generative AI and AI-assisted technologies in the writing process

During the preparation of this work, the authors used ChatGPT to improve the English grammar and language of the manuscript. After using this tool, the authors carefully reviewed and edited the manuscript as needed and take full responsibility for the content of the publication

## Declaration of competing interest

The authors report there are no competing interests to declare.
