# Supplementary material for: Understanding participation and quality of life of university students with probable DCD using structural equation modeling
Source: Braz J Phys Ther. 2026 Oct 5;31(1):101658. doi: 10.1016/j.bjpt.2026.101658 (PMC13672977; doi:10.1016/j.bjpt.2026.101658)
Supplement: Supplementary file 1 [file mmc1.docx]

Supplementary Materials

**Figure A1.** Theoretical Model Explaining Quality of Life (QOL) from Developmental Coordination Disorder (DCD), Executive Functions, Attention Deficit Hyperactivity Disorder (ADHD) and Participation.

QOL

Executive functions

DCD

ADHD

Participation
